# Draft genomes of *Lactobacillus jensenii* UMB0908, UMB1545, and UMB5777 from the urinary tract

**DOI:** 10.1128/mra.01120-23

**Published:** 2024-01-24

**Authors:** Maria Steiling, Briana Jackson, Natalie Stegman, Cerena Sedano, Catherine Putonti

**Affiliations:** 1 Department of Biology, Loyola University Chicago, Chicago, Illinois, USA; 2 Bioinformatics Program, Loyola University Chicago, Chicago, Illinois, USA; University of Maryland School of Medicine, Baltimore, Maryland, USA

**Keywords:** *Lactobacillus jensenii*, urinary tract

## Abstract

*Lactobacillus jensenii* is a member of the female urogenital microbiome. Previous research has identified strains of *L. jensenii* that are capable of inhibiting or killing uropathogens, including *E. coli*. Here, we present the draft genomes of three *L. jensenii* strains collected from urine samples.

## ANNOUNCEMENT

*Lactobacillus jensenii* is a common member of the vaginal and urinary microbiota in healthy females (1–4). It also has been found in the bladders of females with lower urinary tract symptoms (5, 6). Prior research has identified *L. jensenii* strains capable of inhibiting or killing urogenital pathogens (7–9). To further our understanding of bactericidal effects, three *L. jensenii* strains were isolated from urine samples using the enhanced quantitative urine culture method (10). *L. jensenii* UMB908 was isolated from a catheterized urine sample from a pregnant female (LUC IRB #206470), while *L. jensenii* UMB1545 and UMB5777 were isolated from voided urine samples from two different females with type 2 diabetes (LUC IRB #204197) (11). Strains were initially identified as *L. jensenii* by MALDI-TOF, as previously described (5), prior to storage at −80°C.

Strains were streaked on deMan-Rogosa-Sharpe (MRS) agar plates and incubated at 35°C with 5% CO_2_ for 48 hours. After incubation, MRS liquid media were inoculated with a single colony. DNA extraction was conducted using the Qiagen DNeasy Blood and Tissue kit following the manufacturer’s protocol for gram-positive bacteria and quantified using the Qubit fluorometer. DNA was sent to SeqCenter (Pittsburgh, PA, USA). Libraries were created using the Illumina DNA Prep Kit and custom IDT 10 bp unique dual indices with a target insert size of 320 bp. Libraries were sequenced on the Illumina NovaSeq 6000 platform, producing 2 × 151 bp paired-end reads. Demultiplexing, quality control, and adapter trimming were conducted with bcl-convert (v4.1.5). Raw reads were trimmed using BBDuk, which is part of the BBTools suite (v39.01; https://sourceforge.net/projects/bbmap/) with the following parameters: qtrim = rl, ftl = 15, ftr = 135, maq = 20, maxns = 0, and trimq = 20. Trimmed reads then were assembled using SPAdes v15.5 with the --only-assembler option (12). Genome assemblies were annotated using the Prokaryotic Genome Annotation Pipeline v6.6 (13), which confirmed the species designation. Genome sequencing and assembly statistics are listed in Table 1.

**TABLE 1:** Assembly statistics for urinary *L. jensenii* strains

Strain	SRA accession no.	GenBank assembly accession no.	No. of raw reads	No. of contigs	Genome length (bp)	N50 (bp)	Coverage	%GC
UMB0908	SRR26087534	GCA_032377315.1	3,274,986	57	1,703,657	54,358	279.713×	34%
UMB1545	SRR26087532	GCA_032377035.1	4,414,712	44	1,851,201	66,131	320.579×	34%
UMB5777	SRR26087524	GCA_032376755.1	4,509,960	40	1,712,832	67,911	349.565×	34%

Given our interest in their putative antimicrobial potential, the three *L. jensenii* strains were examined for bacteriocins via Bagel4 (14) and secondary metabolites via antiSMASH v7.0.1 (using default parameters) (15). While no bacteriocins were identified, antiSMASH identified a non-ribosomal peptide synthase in the genome assemblies of both UMB0908 and UMB5777, which was previously found to be common among *L. jensenii* strains (16). Further phenotypic characterization is needed to ascertain if this metabolite or others produced by these *L. jensenii* strains confers antimicrobial properties.

## Data Availability

Raw reads and draft assemblies have been deposited in GenBank. Table 1 lists the SRA accession numbers and genome assembly accession numbers.
